# Enhancing detection of common bean diseases using Fast Gradient Sign Method–trained Vision Transformers

**DOI:** 10.3389/frai.2025.1643582

**Published:** 2025-08-06

**Authors:** Upendo Mwaibale, Neema Mduma, Hudson Laizer, Bonny Mgawe

**Affiliations:** ^1^Computational and Communication Science and Engineering (CoCSE), The Nelson Mandela African Institution of Science and Technology (NM-AIST), Arusha, Tanzania; ^2^Life Sciences and Bio-engineering (LiSBE), The Nelson Mandela African Institution of Science and Technology (NM-AIST), Arusha, Tanzania

**Keywords:** bean rust, bean anthracnose, deep learning, Vision Transformers (ViT), adversarial attacks, Fast Gradient Sign Method

## Abstract

Common bean production in Tanzania is threatened by diseases such as bean rust and bean anthracnose, with early detection critical for effective management. This study presents a Vision Transformer (ViT)-based deep learning model enhanced with adversarial training to improve disease detection robustness under real-world farm conditions. A dataset of 100,000 annotated images augmented with geometric, color, and FGSM-based perturbations, simulating field variability. FGSM was selected for its computational efficiency in low-resource settings. The model, fine-tuned using transfer learning and validated through cross-validation, achieved an accuracy of 99.4%. Results highlight the effectiveness of integrating adversarial robustness to enhance model reliability for mobile-based plant disease detection in resource-constrained environments.

## 1 Introduction

Agriculture is vital to Tanzania’s economy, contributing significantly to export revenues and accounting for approximately USD 13.13 billion of the country’s Gross Domestic Product (GDP) in 2023 (Economics, Trading, 2022; Statista, 2023). The Agricultural Census of 2019/2020 revealed that around 7.8 million households (65.3%) are involved in agricultural activities, and out of those, around 5.1 million are exclusively engaged in crop farming only (National Bureau of Statistics, 2019). This highlights the importance of agriculture as the primary occupation for most households in Tanzania (Tryphone and Nchimbi-Msolla, 2010).

The common bean (*Phaseolus vulgaris*) is a crucial crop in Tanzania’s smallholder farming system, serving as both a cash and food crop, and is considered the principal source of dietary protein for more than 75% of rural households (Hillocks et al., 2006; WordBank, 2017). The common bean is also a predominant legume crop, accounting for approximately 78% of cultivated land (Binagwa, 2016). However, common bean farming in Tanzania faces substantial challenges, particularly in managing pests and diseases (Binagwa, 2019; Wolter, 2009). Farmers incur high costs in combating diseases, often without adequate technical support, which leads to ineffective disease control, pollution, and adverse outcomes (Peshin and Dhawan, 2009; Watkins, 2022).

Bean rust and bean anthracnose are among the major diseases that pose significant threats to common bean production for most smallholder farmers in Tanzania (Peshin, 2014). Bean rust is a fungal disease that can severely reduce seed and pod quality, leading to yield losses of up to 100% if not properly managed, particularly in temperatures between 17°C and 25°C with high humidity (Greenlife, 2023; Schwartz et al., 2005). Bean anthracnose, on the other hand, is a seed-borne fungal disease attacking leaves, stems, and pods, often leading to early plant death, especially during cold seasons. Bean anthracnose can cause yield losses ranging from 27 to 86%, depending on weather conditions and management practices (Girma et al., 2022; Buruchara et al., 2010; Masunga et al., 2020; Kadege et al., 2022). Traditional methods, such as crop scouting and visual inspections, are still widely used but have limited capacity for early detection and timely response (Rajabu et al., 2022; Slimani et al., 2023).

To address these limitations, recent advances in deep learning and computer vision have shown promise. These technologies enable automatic identification, classification, and quantification of plant diseases through image analysis, facilitating earlier and more effective disease management (Mohanty et al., 2016; Ferentinos, 2018; Loyani, Loyani et al., 2021). Despite their promise, many models are trained on images from controlled environments, limiting their accuracy in real-world conditions (Alzubaidi et al., 2023). This underscores the need for more diverse, field-acquired datasets to ensure reliable performance in practical settings (Barbedo, 2018).

Beyond dataset limitations, model robustness is also compromised by inherent architectural vulnerabilities in commonly used neural networks. These structural weaknesses have been exploited in crop disease models through adversarial attacks, as reported by Luo and Li (2021). This vulnerability stems from the architectural design of specific neural networks, such as Convolutional Neural Networks (CNNs), Long Short-Term Memory (LSTM) networks, and Rectified Linear Units (ReLU) units, which exhibit linear behavior to facilitate optimization during training (Szegedy et al., 2014). However, this linearity makes these networks vulnerable to adversarial perturbations intentionally designed to exploit this property (Chen and Liu, 2023). When subjected to such perturbations, the performance of these models in accurately classifying plant diseases can be significantly compromised, leading to misclassifications and reduced reliability in real-world applications (Silva and Najafirad, 2020).

Convolutional Neural Networks (CNNs) have long been the foundation of image classification tasks in agricultural applications, particularly in the detection of plant diseases. However, Vision Transformers (ViTs) have recently emerged as a powerful alternative due to their self-attention mechanism, which captures long-range dependencies and subtle patterns in images more effectively than CNNs.

To enhance the performance and robustness of Vision Transformers (ViTs) under noisy, artifact-laden, or adversarial conditions, researchers have adopted adversarial training techniques. This technique exposes models to intentionally modified inputs (called adversarial examples) during training. One of the strongest methods in this category is Projected Gradient Descent (PGD), which iteratively adjusts an image to mislead the model while keeping the changes imperceptible to humans. Similarly, AutoAttack is a composite, automated benchmark that combines multiple adversarial strategies to evaluate a model’s worst-case robustness. While both PGD and AutoAttack provide strong theoretical guarantees, they are computationally expensive and often unsuitable for real-time deployment in low-resource agricultural settings (Ali et al., 2024).

In contrast, the Fast Gradient Sign Method (FGSM) provides a more lightweight adversarial training approach by modifying the input image in a single step (Chang et al., 2018). Although FGSM may be considered weaker than PGD and AutoAttack, studies such as Waghela et al. (2024) have demonstrated that it provides a practical trade-off between robustness and efficiency. Whereas adversarial training in ViTs has been investigated in medical imaging and natural image classification, its application in real-world, field-based plant disease detection remains poorly understood (Naseer et al., 2021).

This study aims to develop a robust plant disease detection model by combining Vision Transformers with FGSM-based adversarial training, tailored for real-world, low-resource agricultural settings. Specifically, this study combines transfer learning using ViT with the FGSM to reduce model vulnerability and enhance detection accuracy. By examining both computational feasibility and adversarial robustness, this research contributes to the growing body of work on deep learning in agriculture, providing practical insights for future applications in resource-constrained farm contexts.

## 2 Materials and methods

### 2.1 Conceptual framework

**Figure 1:**
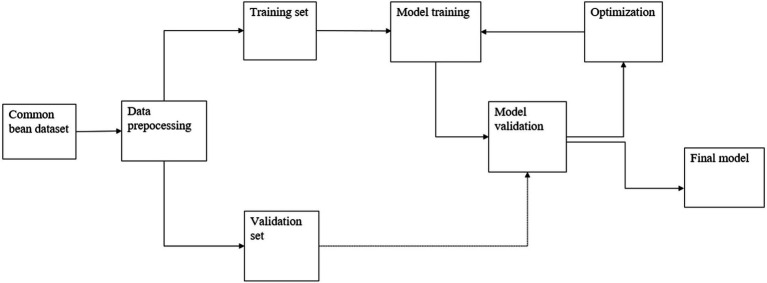
The conceptual framework.

Figure 1 provides an overview of the proposed method from the data collection to validation and delivery of an optimized model. The dataset was collected from farms and then preprocessed; subsequently, it was divided into training and testing sets. The models were then trained on the training set of the dataset, continuously optimized, and then validated using the testing set to obtain an optimized model.

### 2.2 Datasets

A thorough dataset was gathered from bean farms in Tanzania’s Southern Highlands, specifically in the Njombe, Iringa, and Mbeya regions, which were chosen for their high bean output and disease frequency, in order to build a robust model for common bean disease detection (Agro, Techno Strategies, 2018). Using mobile phone cameras under natural field conditions, a total of 59,072 images were gathered over 3 months and published at Zenodo.^1^ The collection comprised healthy leaves as well as those displaying symptoms of anthracnose and rust.

Trained agricultural extension agents with knowledge of plant pathology annotated the images. To identify and classify symptoms at the site, they utilized national diagnostic guidelines from TOSCI (Tanzania Official Seed Certification Institute) manuals and the CIMMYT disease guide. Local agricultural researchers cross-verified the annotation process to lower mislabeling risks, mainly between visually similar diseases. Through achieving a consensus among annotators, uncertain cases were settled and marked for additional examination. An additional class containing unrelated images, sourced from the internet and including noise or artifacts, was added to enhance robustness. This class helped the model learn to identify and appropriately reject inputs that do not correspond to any of the target disease categories. The dataset then consisted of four classes: Healthy, Rust, Anthra, and Images containing noise or artifacts.

### 2.3 Data preprocessing

Before training the model, the dataset underwent a preprocessing process to ensure consistency and compatibility with the model’s input requirements. The images were resized to a uniform dimension of 512×512 pixels, which strikes a balance between preserving important details and maintaining computational efficiency during training. To normalize the pixel values and center the data distribution, each image was divided by 255, scaling the pixel intensities to a range of 0 to 1. This normalization step helps the model converge more quickly during training and reduces the impact of illumination variations (Ioffe and Szegedy, 2015).

**Figure 2:**
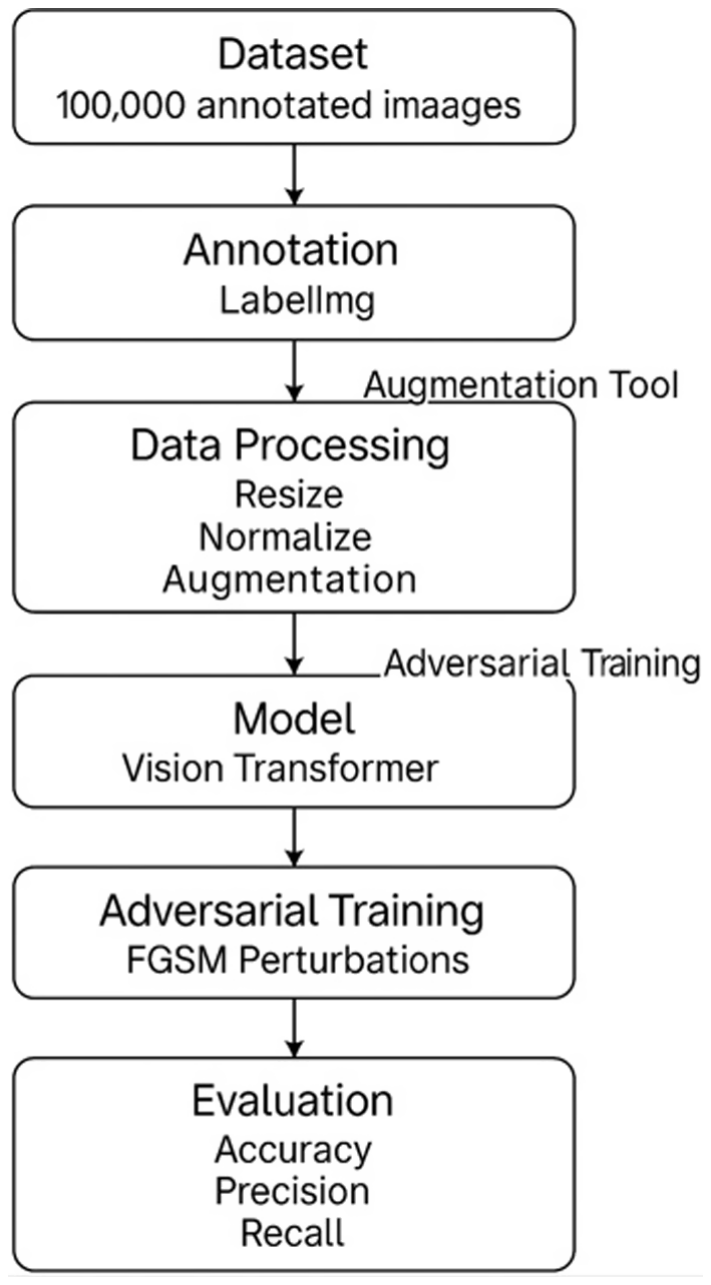
Unified implementation pipeline.

To address the challenges of variability and noise in real-world bean leaf imagery, a unified data preprocessing and augmentation pipeline was implemented, as illustrated in Figure 2. The augmentation process was carried out using the Albumentations library, known for its flexibility and efficiency in computer vision tasks. A modular augmentation suite, including geometric, photometric, and adversarial transformations, was constructed.

To simulate real-world distortions commonly found in farm-acquired images, the augmentation pipeline included transformations such as color jittering, random cropping, Gaussian blur, perspective transformation, elastic deformation, zooming, rotation, and horizontal/vertical flips. To improve robustness against adversarial perturbations, FGSM-based noise was introduced at the input level using custom PyTorch routines. This entire augmentation logic was developed within a reproducible, version-controlled environment to ensure consistency and transparency.

**Figure 3:**
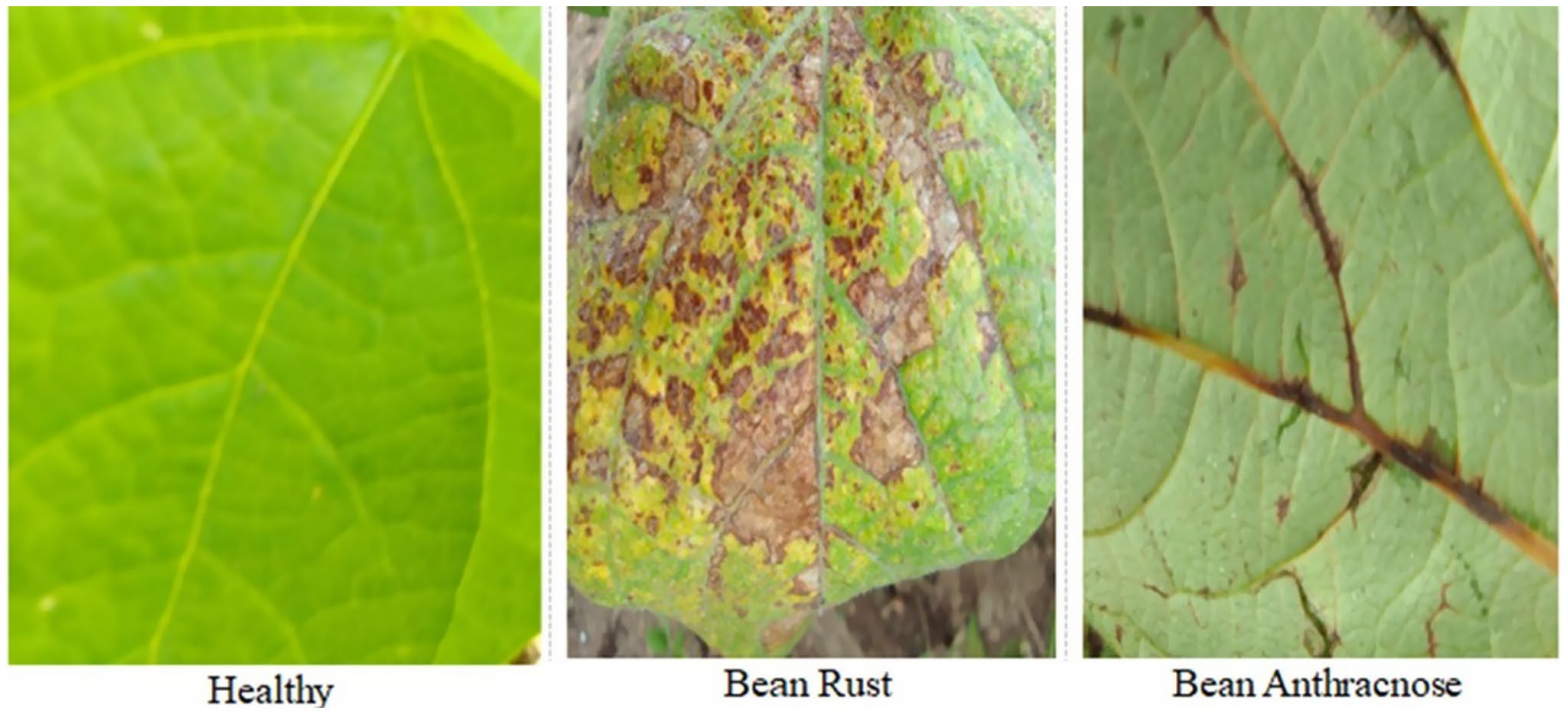
Sample of the common bean leaf images from the dataset.

Image annotation was performed manually using LabelImg, a widely adopted tool for bounding box labeling with deep learning-compatible formats. All transformations and annotations were logged, and the dataset was split into training, validation, and test sets with careful attention to class balance and diversity. These augmentation techniques produced a comprehensive dataset that reflects the variability of real-world field conditions, enhancing the model’s ability to generalize. Figure 3 illustrates examples from the dataset, while Table 1 presents the distribution of the dataset before and after augmentation. To ensure robustness and reduce overfitting, both 5-fold cross-validation and an 80/20 hold-out test split were applied.

**Table 1:** Dataset distribution before and after augmentation.

Class	Initial images	Augmented images
Images including noise or artifacts	17,531	25,000
Rust	22,198	25,000
Anthracnose	20,000	25,000
Healthy	24,973	25,000
Total	84,072	100,000

#### 2.3.1 Adversarial noise injection

The Fast Gradient Sign Method (FGSM) is an efficient technique for generating adversarial examples by introducing subtle, structured perturbations to input data in a way that intentionally misleads a model. Initially proposed by Goodfellow (2015) FGSM is based on the insight that deep learning models, particularly those using ReLU activations, are often too linear in high-dimensional spaces, making them susceptible to small perturbations that can lead to misclassification.

According to Goodfellow et al. (2020) the FGSM algorithm computes adversarial examples by adjusting the input image x in the direction of the gradient of the loss function J(*θ*, x, y).

x_adv=x+εsign[∇_xJ(θ,x,y)]

Where:

- x_adv is the adversarial image,
- ε is a small scalar that controls the size of the perturbation,
- ∇_x J(θ, x, y) is the gradient of the loss to the input,
- sign(·) indicates the sign function,
- θ represents the model parameters,
- y is the actual label.

FGSM perturbs the image in the direction that increases the model’s loss, producing a visually similar image that can deceive the classifier if it has not been trained to be robust (Tramèr et al., 2018).

In the context of agricultural disease detection using images captured in uncontrolled environments, such as farm fields, these perturbations simulate real-world image degradations caused by environmental noise, motion blur, low-light conditions, unintended hand motion, or poor camera quality. By training the model with FGSM-augmented images, the model learns to resist these small adversarial changes, thus enhancing its reliability and robustness during deployment in practical, resource-constrained settings.

To enhance the model’s resilience against adversarial inputs and ensure robust performance under real-world variations, the FGSM was implemented. This forced the model to focus on the essential discriminative features rather than overfitting to minute details that may be corrupted by noise. As demonstrated by Xu et al. (2019), FGSM enhances model robustness by exposing it to realistic adversarial scenarios.

#### 2.3.2 Perspective transformation

The real-world deployment of leaf disease detection models often involves mobile device cameras held at various angles. To prepare the model for this variability, perspective transformation was applied to simulate image captures from different viewpoints. As discussed by Ahmad et al. (2023), such transformations mimic real camera tilts and slants, thereby training the model to generalize better under non-frontal, off-axis imaging conditions. This helps ensure consistent and accurate performance when used in the field, especially by farmers with limited photography experience.

#### 2.3.3 Color jitter

Outdoor image capture is subject to unpredictable lighting conditions such as bright sunlight, shadows, or cloudy skies. To simulate real-world conditions and enhance the model’s adaptability, color jitter was applied to randomly adjust the image’s brightness, contrast, saturation, and hue. This technique, recommended by Howard et al. (2017), diversifies the lighting spectrum encountered during training, allowing the model to maintain accuracy regardless of the environmental lighting conditions during image acquisition. This is particularly important in resource-constrained agricultural environments where lighting control is not feasible.

#### 2.3.4 Rotation and flips

To increase the model’s robustness to the orientation of leaves in captured images, random horizontal and vertical flips, as well as 90°, 180°, and 270° rotations, were applied (Shorten and Khoshgoftaar, 2019). It has been shown that such transformations are effective in preventing the model from becoming orientation-sensitive. This augmentation ensures that disease symptoms can be accurately detected, regardless of how the leaf is positioned during capture, a vital consideration when relying on non-expert users, such as farmers or extension officers.

#### 2.3.5 Random zooming

To simulate the varying distances at which users may take photos of leaves, random zoom transformations were introduced. This technique exposes the model to different scales of the same object, improving its ability to detect disease features across zoom levels. According to the work of Cubuk et al. (2019), scale-aware models are crucial for field-based applications, particularly when users unintentionally vary the distance between the camera and the leaf.

#### 2.3.6 Gaussian blur

**Figure 4:**
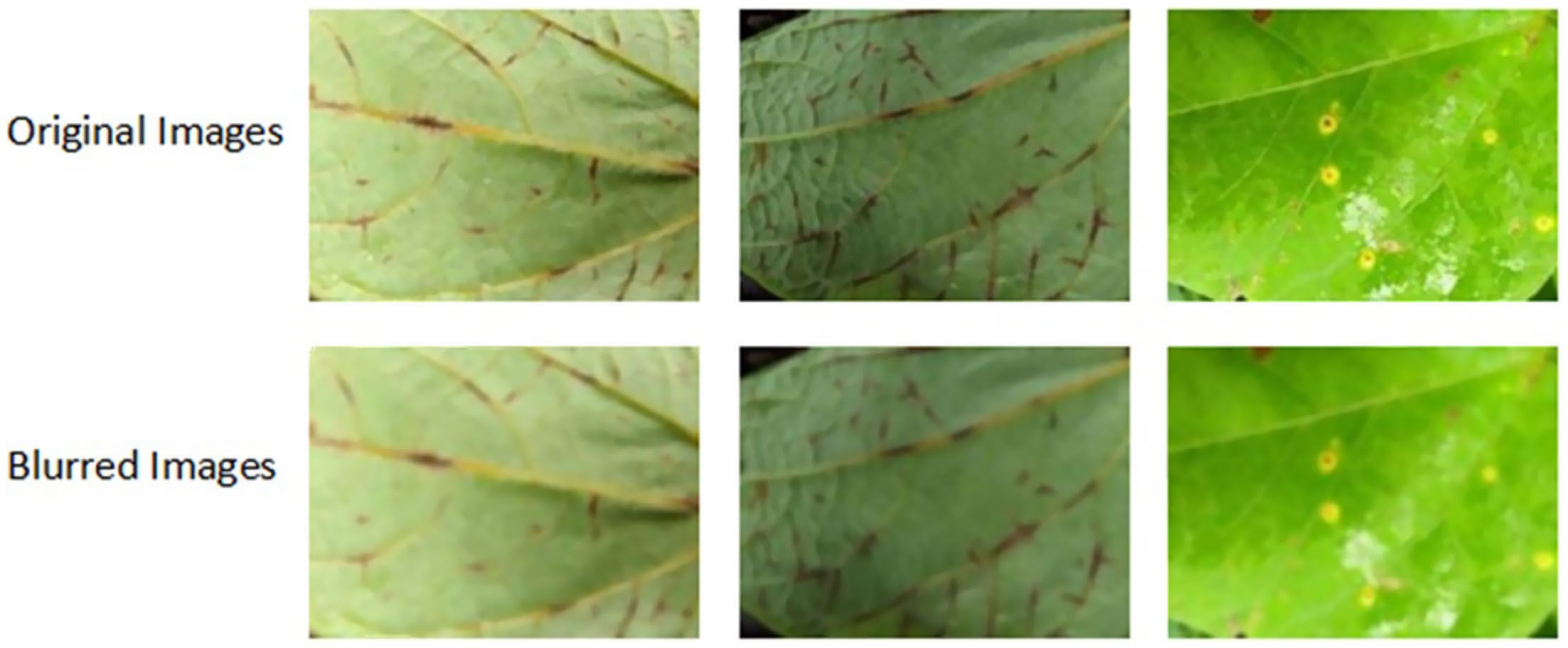
Examples of original images and images with Gaussian blur mimicking a low phone camera, typical of mobile phones used by farmers in the field.

To account for the common issue of motion blur or camera focus imperfections, especially in handheld mobile photography, Gaussian blur was applied to a subset of the images. This technique subtly blurs the image, simulating the effects of camera shake or low-light focusing errors. Lim et al. (2019) suggest that incorporating blur in training data increases the model’s tolerance to low-fidelity inputs without degrading performance. Figure 4 shows sample dataset images of original images and images with a Gaussian blur.

#### 2.3.7 Random cropping

Field images may contain only parts of leaves due to improper framing, occlusion, or zooming. Random cropping was applied to simulate partial visibility and train the model to detect disease symptoms even when the entire leaf is not visible. Rebuffi et al. (2021) highlighted this approach as beneficial for learning local features and improving detection accuracy in unpredictable field conditions.

#### 2.3.8 Elastic transformation

Leaves naturally vary in shape due to genetic, environmental, and maturity differences. To simulate such realistic morphological diversity, elastic transformations were applied. This technique introduces small spatial deformations that mimic stretching or warping, helping the model generalize to non-uniform leaf shapes. Snyder et al. (2015) demonstrated the effectiveness of this method in improving resilience to biological variation.

### 2.4 The ViT model

**Figure 5:**
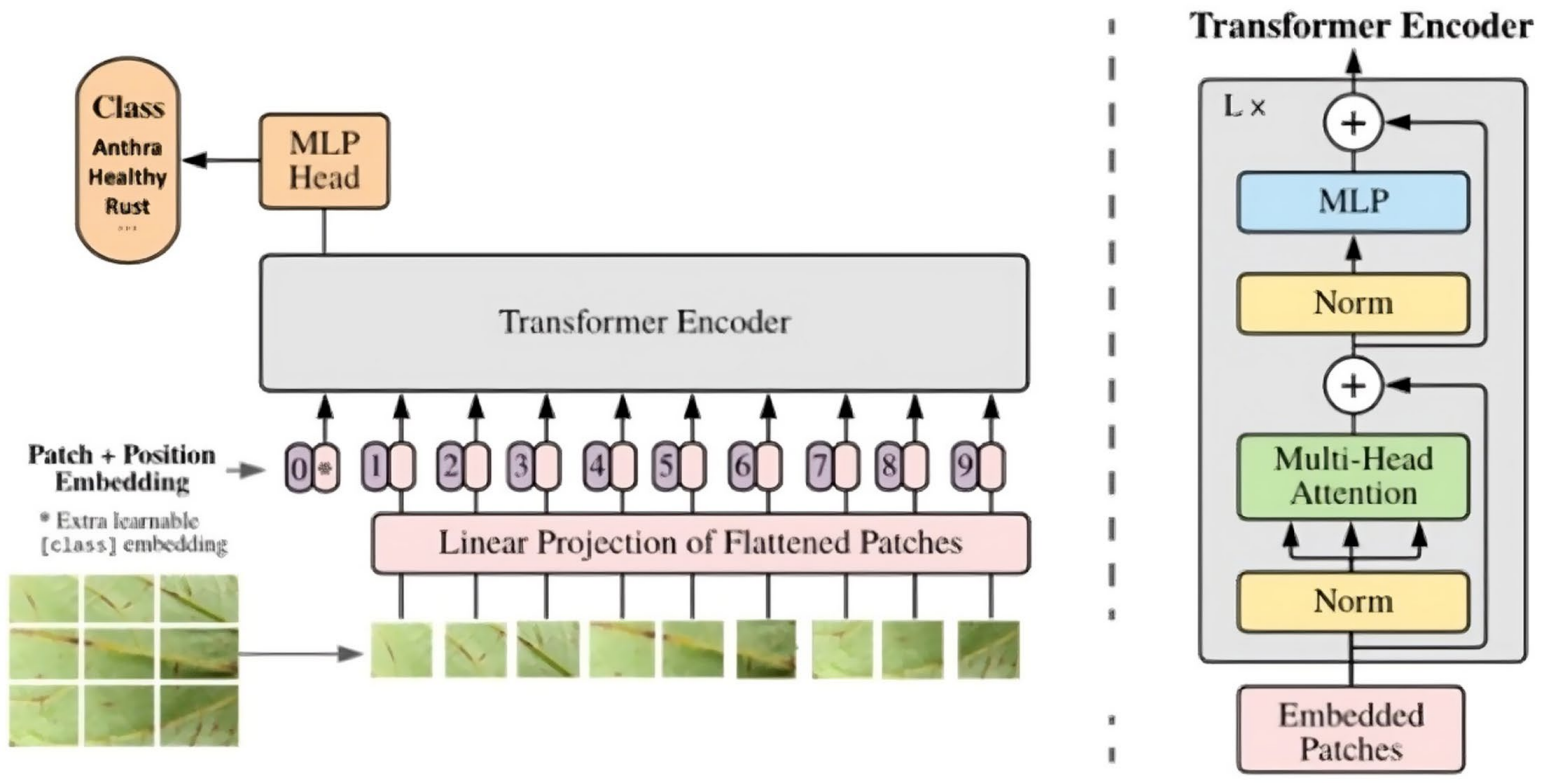
Vision Transformer (ViT) architecture (Dosovitskiy et al., 2021).

The Vision Transformer (ViT) is a state-of-the-art deep learning model that achieves remarkable performance in various computer vision tasks, including image classification, object detection, and semantic segmentation (Dosovitskiy et al., 2021). The ViT architecture is based on the self-attention mechanism, which allows the model to capture global dependencies and learn effective representations from image data (Zhai et al., 2022). Figure 5, inspired by Dosovitskiy et al. (2021), illustrates the architecture of a ViT model adapted for classifying common bean leaf diseases.

The input to the model is a common bean leaf image, consisting of 512×512 pixel images, which is first divided into a sequence of 16×16 patches. This patching process enables the model to capture the local features and spatial relationships of the leaf image, resulting in a total of 1,024 non-overlapping patches per image. Before being fed into the Transformer Encoder, image patches undergo a linear projection to map them to a 768-dimensional embedding space, and a learnable positional embedding is added to preserve the spatial structure of the input, thereby enabling more expressive representations. The patches were passed through 12 Transformer encoder layers, each comprising a multi-head self-attention mechanism with 12 heads, followed by a feed-forward layer with a hidden dimension of 3,072 units. The Multi-Head Attention block, with multiple attention heads, helps capture diverse features and relationships between patches, thereby enhancing the model’s focus on relevant parts for disease classification.

The final output passes through a classification head, a Multi-Layer Perceptron (MLP), which maps the learned representations to specific disease classes, such as Anthracnose, Healthy, or Rust. The model uses supervised learning to associate representations with disease labels, optimizing to minimize classification errors. The Vision Transformer (ViT) architecture effectively captures local and global features, making it well-suited for plant disease classification, particularly for common bean leaves. Training on diverse datasets, the model generalizes well to unseen samples, improving disease detection accuracy in real-world scenarios. Additionally, adversarial training enhances the ViT model’s robustness and generalization.

### 2.5 Model training

**Figure 6:**
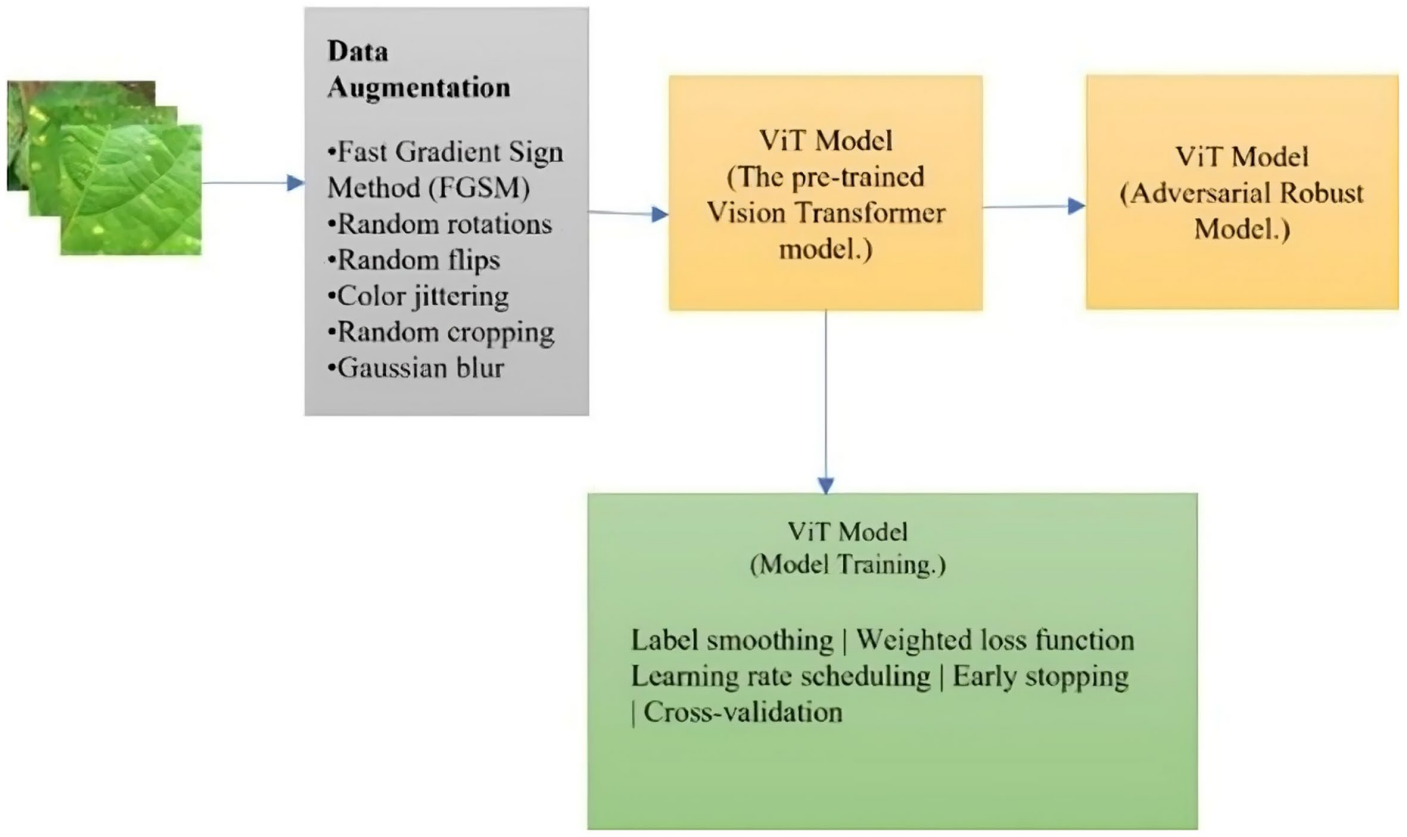
The conceptual framework for the training workflow.

The dataset passed through various stages during model training, as illustrated in Figure 6. The dataset was enhanced using a variety of augmentation techniques. The methods included the incorporation of the Fast Gradient Sign Method (FGSM) and random transformations such as rotations, flips, and perspective transformations. FGSM was employed as the adversarial training method due to its computational simplicity and ability to simulate real-world perturbations such as image blur or lighting changes (Waghela et al., 2024). Unlike PGD or AutoAttack, which require multiple backward passes, FGSM is suitable for rapid augmentation and compatible with on-device learning. A comparative experiment was conducted using PGD (3 iterations, *ε* = 0.03), which demonstrated slightly improved robustness but required three times more training time and greater memory usage, making FGSM a more practical option for low-resource settings (Saleem et al., 2025). Other augmentation methods applied were color jittering, random cropping, and Gaussian blur. Label smoothing was employed as a regularization technique to prevent the model from becoming overconfident in its predictions (Müller et al., 2019). To mitigate the impact of noisy labels and outliers in the dataset, a weighted loss function was utilized to assign different weights to samples based on their reliability (Wang et al., 2020). The weights were calculated by adding adversarial noise, which was injected by using FGSM during training with epsilon set to 0.05.

The model used was ViT-Base (ViT-B/16), which has 12 layers, 12 attention heads, and 768 hidden dimensions. To adapt ViT for the specific task of common bean disease detection, the model was fine-tuned through transfer learning. The model was initialized with pre-trained weights from the ImageNet dataset, providing a solid foundation for feature extraction. The model was trained for six epochs, given computational restrictions and early convergence trends seen in pilot runs. The powerful feature transfer most likely caused this performance plateau by limiting additional accuracy gains outside of the early epochs. During training, the learning rate was dynamically adjusted using the 1-cycle learning policy, with a default peak learning rate of around 0.003 and early stopping with a patience of 10 epochs if the validation loss did not improve. The optimizer used was AdamW with *β*₁ = 0.9, β₂ = 0.999, and a weight decay of 0.01. A batch size of 64 was used from the available GPU memory. Hyperparameter tuning was performed using a grid search strategy on learning rate (0.001–0.005), patch size (16, 32), and dropout rate (0.1–0.3), selecting the best configuration based on validation F1-score. These were done to prevent overfitting and ensure the model’s convergence. Cross-validation was employed to evaluate the model’s performance and ensure its robustness across various subsets of the data.

Performance metrics were averaged across folds to ensure consistent evaluation across different subsets. The model’s performance was evaluated using various metrics, including loss, accuracy, error rate, precision, recall, and F1 score. Table 2 summarizes the hyperparameters and value(s) used for training the ViT model.

**Table 2:** ViT model training hyperparameters.

Parameter(s)	Value(s)
Epoch	6
Optimizer	Adam
Learning rate	0.003
Evaluation metric	Accuracy, Precision, Recall, F-measure
Loss	Categorical cross-entropy

### 2.6 Experimental setup

The study used the Tesla V100 GPU, which features NVIDIA Volta Architecture with 7 TFLOPS of double-precision Performance and 14 TFLOPS of Single-Precision Performance. It has 5,120 CUDA Cores and 640 Tensor Cores, 32GB of high-bandwidth memory (HBM2) VRAM, and a PCIe 3.0 ×16 Interface for fast data transfer. The training was completed in approximately 6 h for six epochs, with a batch size of 64. The peak GPU memory usage averaged 27 GB, and the inference speed was recorded at 43 ms per image, supporting real-time deployment in field conditions.

## 3 Results

### 3.1 Results

**Figure 7:**
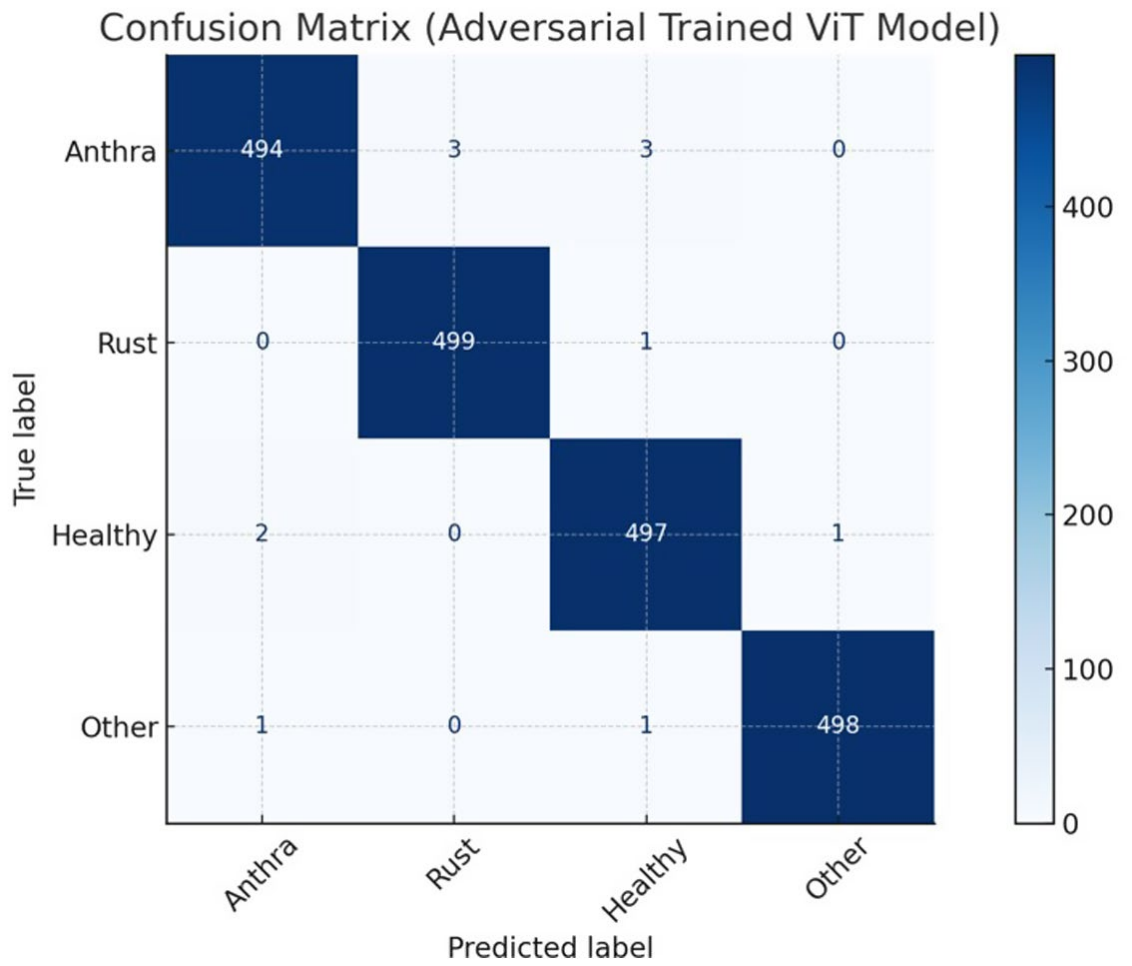
Confusion matrix for adversarial trained ViT model.

**Figure 8:**
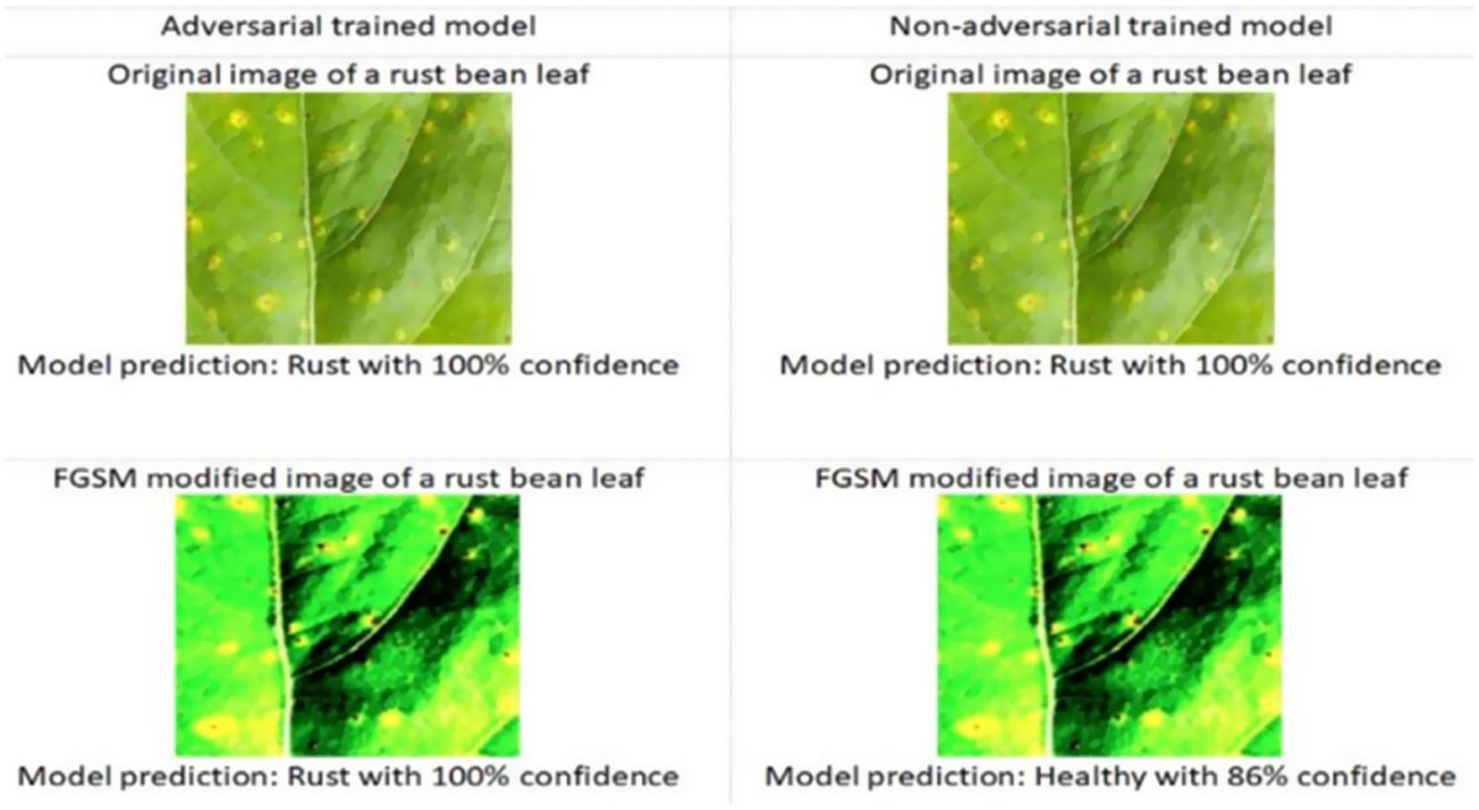
Prediction of adversarial versus non-adversarial models.

**Figure 9:**
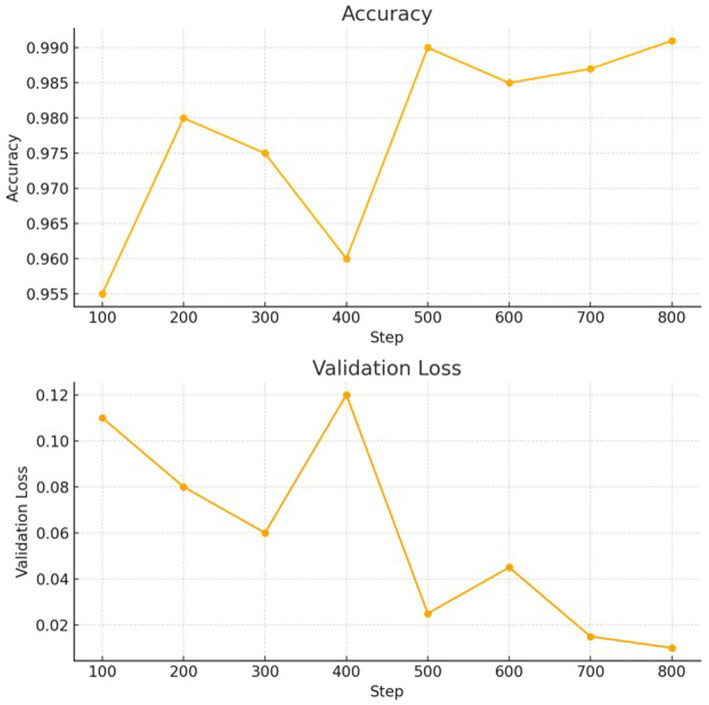
Performance (validation accuracy & validation loss) of the adversarial trained ViT model.

**Figure 10:**
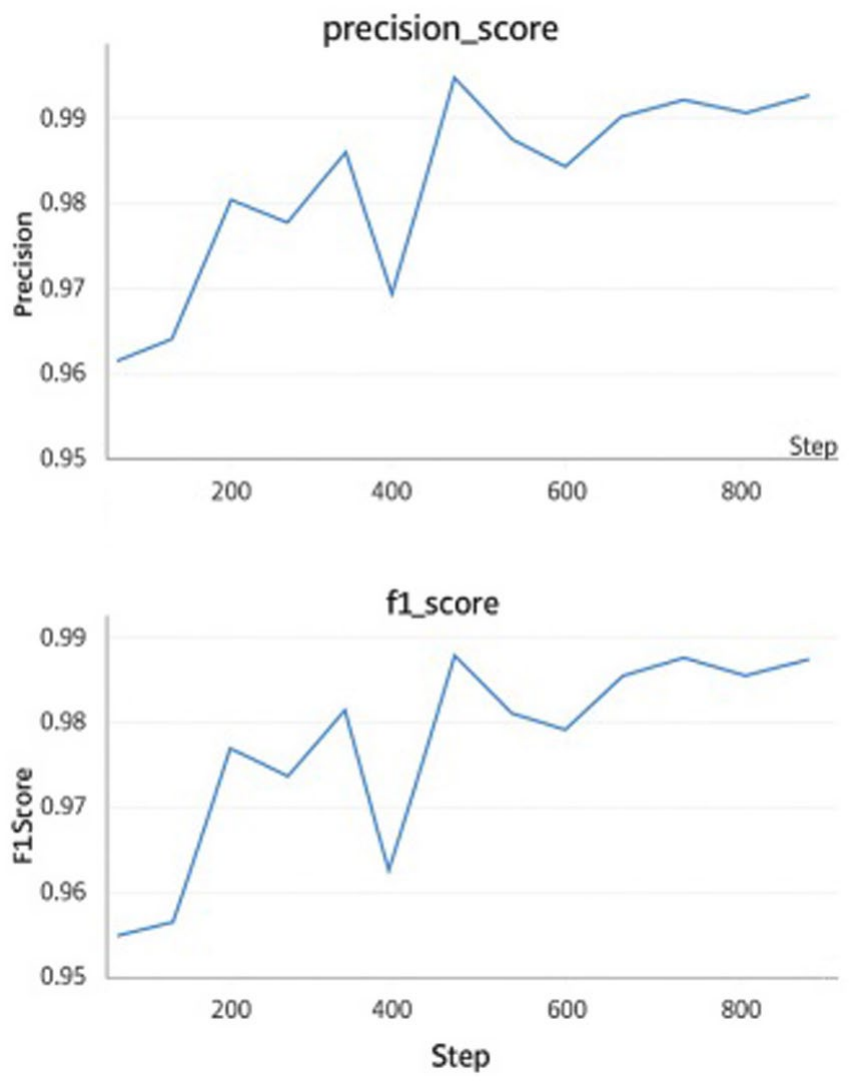
Performance (Precision score &F1 score) of the adversarial trained ViT model.

The adversarially trained Vision Transformer (ViT) model demonstrated superior performance on the test set compared to the adversarially trained CNN and non-adversarially trained ViT model. Precision, recall, and F1 scores were computed using micro-averaging. Due to the model’s strong performance across balanced classes and minimal classification errors, these metrics converged to the same value. The robustness was evaluated using adversarial accuracy under varying FGSM perturbations (*ε* = 0.01 to 0.05). As ε increased, accuracy decreased moderately, but the FGSM-trained model consistently outperformed the baseline. Applying FGSM perturbations at the input layer resulted in a + 1.2% improvement in test accuracy compared to perturbing intermediate encoder layers. Table 3 presents a detailed comparison of the results for the adversarially trained ViT model with the adversarially trained CNN and non-adversarially trained Vision Transformer (ViT) models. Table 4 presents the per-class evaluation metrics of the adversarial-trained ViT model. Figure 7 illustrates the confusion matrix, while Figure 8 illustrates an example of predictions made by the two models (adversarial ViT and non-adversarial ViT). Figure 9 shows the validation accuracy and loss curves of the adversarially trained ViT model. The accuracy trend increases, reaching a peak of 99.1%, while the validation loss gradually decreases, with slight fluctuations. This pattern indicates stable training and good generalization, suggesting that the model effectively learned relevant features without overfitting. Notably, after step 500, the validation accuracy remains high while the loss continues to decrease, confirming the convergence and robustness of the training process under adversarial conditions. Figure 10 shows the Precision score and F1 score curves of the adversarially trained ViT model. The precision steadily increases from approximately 0.955 to over 0.99 as training progresses. This trend suggests that the model is becoming increasingly accurate in correctly identifying positive disease cases while minimizing false positives. The F1 score, which balances precision and recall, also improves consistently, reaching a value of around 0.985. The slight fluctuations observed around step 400 may reflect the model adjusting to complex samples; however, the overall improvement suggests effective learning and better generalization.

**Table 3:** Performance metrics of the ViT model on the test set.

Metric	Adversarial trained model	Adversarial CNN-trained model	Non-adversarial trained model
Accuracy	0.9940	0.9765	0.9740
Loss	0.0105	0.0504	0.0709
Error rate	0.0061	0.0235	0.0260
Precision score	0.9940	0.9752	0.9740
Recall	0.9940	0.9750	0.9740
F1 score	0.9940	0.9751	0.9740

**Table 4:** Per-class evaluation metrics.

Class	Precision	Recall	F1-score	Support
Anthra	0.9940	0.9880	0.9910	5000.0000
Rust	0.9940	0.9980	0.9960	5000.0000
Healthy	0.9900	0.9940	0.9920	5000.0000
Other	0.9980	0.9960	0.9970	5000.0000
Accuracy	0.9940	0.9940	0.9940	5000.0000
Macro avg	0.9940	0.9940	0.9940	5000.0000
Weighted avg	0.9940	0.9940	0.9940	20000.0000

### 3.2 Comparative results with other related works

The model efficiency results from other related studies were compared to those obtained in this work. The results of this study moderately align with those of earlier works, suggesting both convergence and divergence in findings (Table 5).

**Table 5:** Comparative results with other related works.

Crop diseases	Dataset	Model architectures	Classification task	Reference	Accuracy
Common bean leaf diseases	1,766 collected images of bean leaves	GoogleNet	Multi-class (4 classes)	Walle et al. (2024)	96%
Bean leaf diseases	1,295 collected images of bean leaves	Densenet121	Multi-classification (3 classes)	Abed et al. (2021)	98.31%
Bean leaf diseases	iBean	EfficientNetB6	Multi-classification (2 classes)	Singh et al. (2022)	91.74%
Several common bean diseases	PlantVillage	ViT	Multi-classification	Borhani et al. (2022)	97%
Bean rust and anthracnose	84,072 collected images augmented to 100,000 images of common bean leaves	ViT	Multi-classification (4 classes)	Proposed method	99.40%

## 4 Discussion

The study developed two Vision Transformer (ViT) models, one trained with FGSM adversarial augmentation and the other without, to detect common bean rust and anthracnose diseases. The adversarially trained ViT significantly outperformed its non-adversarial counterpart across all primary evaluation metrics, including accuracy, precision, recall, F1-score, and loss reduction, affirming the hypothesis that adversarial robustness enhances model performance under real-world noise.

**Figure 11:**
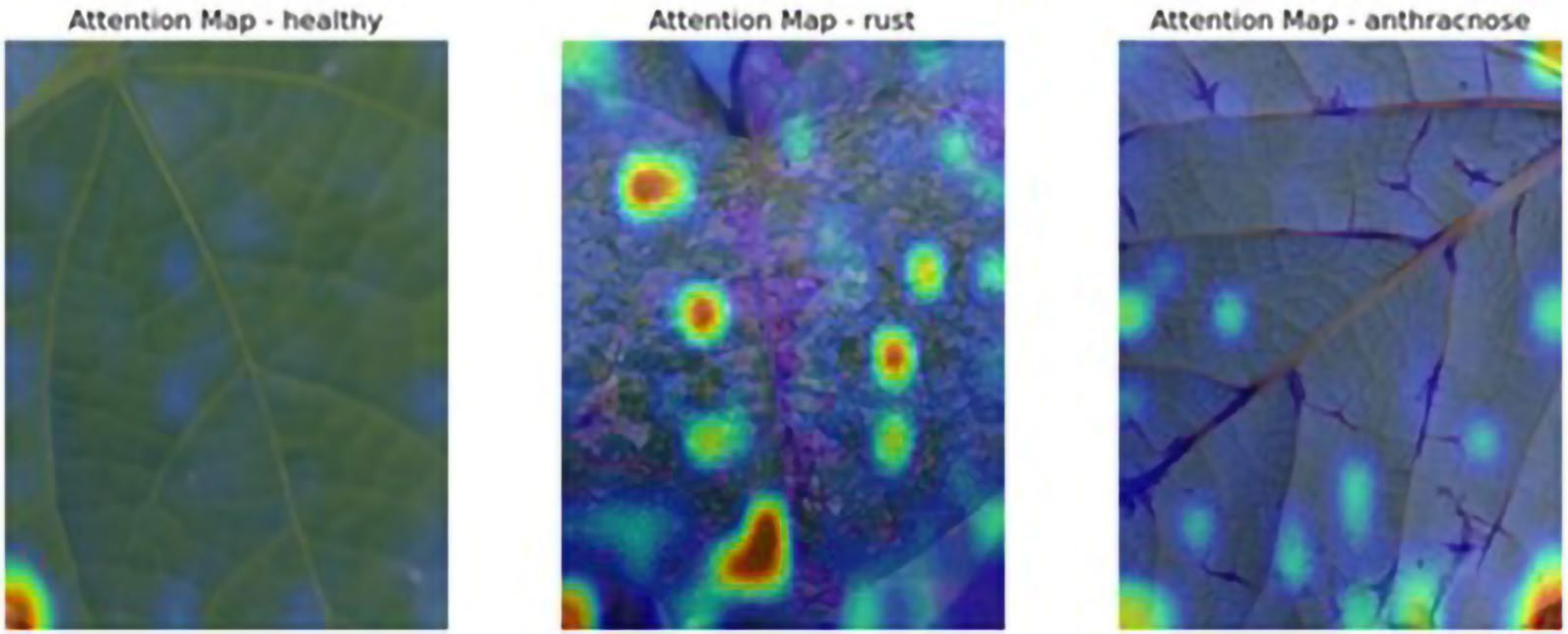
Attention maps.

To evaluate the added value of the proposed adversarially trained ViT model, a comparative analysis was conducted against a CNN baseline trained under the same FGSM-based adversarial training regime. CNNs have traditionally demonstrated strong performance in plant disease tasks due to their ability to extract local features; however, they also exhibit inferior generalization under FSGM perturbations. The ViT model achieved higher accuracy and F1-score compared to the CNN model, indicating its enhanced ability to capture long-range dependencies and complex patterns in leaf textures. This validates that adversarially trained ViTs outperform even robust CNN architectures in challenging agriculture environments. These findings underscore the importance of incorporating transformer-based architectures in agricultural image analysis tasks, where data variability and environmental noise are prevalent. This comparison also addresses the concern regarding the absence of baseline models, demonstrating that the proposed ViT not only improves performance but also outperforms comparable CNNs under identical adversarial conditions. The ViT model’s attention maps offered insights into its decision-making, enhancing its interpretability and trustworthiness. This makes it a valuable tool for farmers, providing reliable disease detection even with limited imaging equipment. Figure 11 illustrates attention maps, which visualize the parts of the image that the model focused on.

To evaluate the generalizability of the proposed model beyond the curated dataset, an additional test was conducted using an independent set of real-world common bean leaf images collected directly from farms under natural conditions. This test set included leaves with varying lighting conditions, mixed infections, signs of abiotic stress, and pest damage conditions that commonly occur in the field but are underrepresented in controlled datasets. Trained agricultural extension agents used TOSCI and CIMMYT guidelines to assign labels to images showing mixed infections and stress symptoms. Each image was categorized based on field diagnosis records and observable symptom overlap. In cases where multiple diseases co-occurred or stress symptoms were present, the dominant visible symptom guided the labeling process. Where ambiguity arose, expert consensus and verification by local agricultural researchers ensured consistency in annotations. Although the model was not explicitly trained to detect multiple diseases within a single image, its predictions on such cases were independently examined during evaluation. The model exhibited high predictive accuracy across these challenging cases, demonstrating robustness in uncontrolled field conditions. Figures 9, 10 illustrate a gradual increase in accuracy and F1-score, accompanied by a slow decrease in loss over the training epochs, indicating improved learning, generalization, and limited overfitting, as shown by the descending validation loss.

These findings are consistent with Goodfellow (2015), who initially proposed the FGSM as a way to generate adversarial examples that force models to learn more invariant and robust features. Studies like You et al. (2023), demonstrated improved generalization in plant disease classifiers using adversarial perturbations. In agricultural contexts, where input images are often noisy and inconsistent, this robustness becomes not just beneficial but essential. Unlike Singh et al. (2022), who reported an accuracy of 91.74% using EfficientNetB6 on clean datasets, the proposed model achieved an accuracy of 99.4% on noisy, field-collected data. This highlights the superiority of adversarial training under realistic deployment conditions. These results align with Gomez et al. (2024), who emphasized the importance of robust augmentation techniques for object detection tasks in complex farm environments.

Compared to prior ViT-based models, such as those used in Borhani et al. (2022), the proposed model introduces FGSM augmentation as a key enhancement. Expanding the comparison to Kumar (2024), who utilized a lightweight DeiT-Tiny transformer on clean data, our full-scale ViT (86 M parameters) was trained and tested on unstructured, noisy images, demonstrating robustness despite the higher computational cost. Nevertheless, since FGSM is a single-step perturbation method, it might not fully capture the range of adversarial noise that exists in the real world. To further increase robustness, future research should investigate more powerful multi-step techniques, such as PGD or diverse augmentation.

Inevitable trade-offs were observed in this approach. While adversarial training enhances robustness, it can increase training time and introduce a risk of overfitting to adversarial patterns rather than natural variance. Additionally, this ViT model, although high-performing, requires more memory and compute resources than CNN-based architectures. The ability to scale is limited by this constraint, particularly in low-resource environments where devices may not be able to support such models without significant optimization. Nevertheless, the model exhibits potential for mobile deployment, with benchmarked inference speed of less than 300 ms on a Snapdragon 865 device.

Geographic constraints are one of the study’s limitations; the dataset used reflects Tanzanian field conditions and may not apply to other regions or crop types. Significant differences in leaf morphology, disease symptom expression, and environmental noise across different ecosystems could affect classification accuracy. Furthermore, the broader impacts of deploying AI disease detection tools for smallholder farmers necessitate that systems consider digital literacy, user trust, and equitable access. Participatory design, involving local farmers, extension officers, and policymakers, will be essential to ensure the responsible deployment. Future iterations of this project should address ethical issues, particularly those related to algorithmic transparency and data privacy. To reduce the risk of misdiagnosis and enhance stakeholder trust, model calibration methods, confidence scoring, and explainable AI modules should be employed.

## 5 Conclusion and future work

This study developed a Vision Transformer (ViT) model enhanced with Fast Gradient Sign Method (FGSM) adversarial training for the early detection of common bean rust and anthracnose under field conditions in Tanzania. The proposed model demonstrated high classification accuracy, precision, recall, and F1-score, outperforming both the adversarially trained CNN and the non-adversarial ViT model. These results highlight the importance of incorporating adversarial robustness into vision-based plant disease classifiers, particularly in noisy, real-world settings. To ensure the reliability of the findings, a 5-fold cross-validation was performed, and classification results were reported as mean ± standard deviation. The test analyzed the accuracy scores of each fold to statistically support the performance difference between the adversarial and non-adversarial ViT models. Additionally, the results confirmed the robustness of the proposed strategy by showing that the performance increase from FGSM training was statistically significant (*p* < 0.05).

Despite these promising results, the study has certain limitations, including the exclusion of comparative analysis with alternative transformer variants, which limits the assessment of the ViT model’s broader effectiveness. Future work should focus on expanding the comparative framework to include other deep learning models, such as EfficientNet, DenseNet, and hybrid CNN-Transformer architectures, under similar adversarial training schemes. Furthermore, interpretability can be enhanced by integrating techniques such as Grad-CAM to illustrate model decision-making pathways. Expanding the dataset to capture a more diverse range of bean cultivars, disease stages, and regional variations will enhance the generalizability of the findings. Finally, deploying the model in lightweight mobile or edge-based platforms and validating its usability with farmers and agricultural experts will be essential for real-world application.

## Data Availability

The datasets presented in this study can be found in online repositories. The names of the repository/repositories and accession number(s) can be found at: https://zenodo.org/api/records/8286126/files-archive.

## Footnotes

^1^ https://zenodo.org/api/records/8286126/files-archive
